# Anti-obesity and immunomodulatory effects of oil and fermented extract dried from *Tenebrio molitor* larvae on aged obese mice

**DOI:** 10.1080/19768354.2024.2374547

**Published:** 2024-07-13

**Authors:** Seul-Ki Mun, Chang Joo Jang, Semi Jo, Si-Hyoun Park, Hyun Bo Sim, Sonny C. Ramos, Hyeongyeong Kim, Yu-Jeong Choi, Dae-Han Park, Kyung-Wuk Park, Beom-Gyun Jeong, Dae Heon Kim, Kyung-Yun Kang, Jong-Jin Kim

**Affiliations:** a Department of Biomedical Science, Sunchon National University, Suncheon, Republic of Korea; b R&D team, Suncheon Research Center for Bio Health Care, Suncheon, Republic of Korea; c CCRIPO Inc., Daejeon, Republic of Korea

**Keywords:** Aging, obesity, *Tenebrio molitor*, insect consumption, immunomodulatory effects

## Abstract

Preventing disease and maintaining the health of the elderly are crucial goals for an aging population, with obesity and immune function restoration being of paramount importance. Obesity, particularly visceral obesity characterized by excessive fat accumulation around the abdominal organs, is linked to chronic conditions such as diabetes, hypertension, cardiovascular diseases, and immune dysfunction. Globally, obesity is considered a disease, prompting significant research interest in its treatment. Therefore, it is essential to explore potential therapeutic and preventive strategies to address obesity and the decline in immune function brought about by aging. *Tenebrio molitor* larvae (TML), commonly known as ‘mealworms,’ are rich in unsaturated fatty acids, including oleic and linoleic acids, and essential amino acids, such as isoleucine and tyrosine. In this study, we aimed to investigate the effects of the consumption of TML oil and mealworm fermented extract (MWF-1) on obesity and immunological changes in aged obese mice. Our data showed reduced body fat in 23-week-old C57BL/6 mice fed processed TML products for 6 weeks. Additionally, the characteristically high levels of serum triglycerides decreased by treating with TML oil. The immune responsiveness results confirmed an increase in B cells by treating with MWF-1, while cytokine levels (interferon-gamma, tumor necrosis factor-alpha, interleukin-2, and −6) were restored to levels similar to young mice. These results suggest that TML oil and MWF-1 are promising dietary supplements for addressing obesity and restoring immune function.

## Introduction

The aging population is a new global issue, and active research has been conducted on various dietary supplements aimed at healthy aging (Wahlqvist and Saviage 2000; Warren 2010; Kim et al. 2016; Song et al. 2020). Changes in metabolism and decreased physical activity in old age are associated with various health issues, including obesity and immune dysfunction (Aiello et al. 2019; Feehan et al. 2021; Kwon et al. 2023). As aging progresses, there is a reduction in skeletal muscle mass and strength, a shift in fat distribution from subcutaneous to visceral fat storage, and an increase in the levels of cholesterol and triglycerides in the bloodstream (Berry et al. 2017; Zoico et al. 2019; Jung et al. 2022). Increased cholesterol levels are associated with the risk of hypertension, stroke, and coronary artery disease (Matsuzawa et al. 2011; Yokokawa et al. 2021). Moreover, the increase in intra-abdominal fat due to obesity excessively stimulates adipose tissue, leading to the secretion of inflammatory cytokines such as leptin, tumor necrosis factor-alpha (TNF-α), and interleukin (IL-6) by fat cells (Franceschi et al. 2002; Na et al. 2015; Frasca et al. 2017; Ghanemi et al. 2021; Frasca et al. 2023; Kang and Lee 2024). Overproduction of these cytokines induces insulin resistance and contributes to metabolic disorders such as hypertriglyceridemia and hypertension (Ahima 2009; Kwon et al. 2023). Obesity was classified as a disease by the World Health Organization (WHO) in 1996, and many approaches to treating it are currently being researched (Upadhyay et al. 2018).

Immunity is the body’s defense system that protects against infections and diseases and is essential for maintaining a healthy life throughout the lifespan (Maggini et al. 2009). Among immune cells, B cells are responsible for antigen recognition, antigen presentation, immune regulation, antibody production, and humoral immune responses associated with antibodies (Yeo et al. 2001). T cells can recognize and eliminate infected cells and induce cellular immune responses by obtaining information about antigens from antigen-presenting cells (APCs) that use major histocompatibility complex (MHC) molecules (Sim and Lee 2003). IL-6 acts as a signaling cytokine that informs the body about tissue damage or infection (Mihara et al. 2012; Kang et al. 2017; Cha and Hong 2024). It also has multiple functions in inducing T-and B-cell differentiation (Yeo et al. 2001; Frasca et al. 2020). TNF-α regulates the activation of inflammatory immune cells, while interferon-gamma (IFN-γ) induces the activation of cellular immunity such as natural killer (NK) cells, adaptive immune system CD4+ helper and CD8+ cytotoxic T cells, and macrophages (Kishimoto et al. 1994; Balkwill and Burke 1998; Sim and Lee 2003; Mihara et al. 2012; Frasca et al. 2020). IL-2 regulates T cell survival, activation, and differentiation (Carding et al. 1991; Schluns and Lefrançois 2003). However, as aging progresses, the immune system is affected by a reduction in the number of white blood cells capable of producing appropriate amounts of cytokines and responding to new antigens (Rego et al. 1998; Blanco et al. 2018). In addition, memory T cells that recall previously encountered antigens may exhibit a low response to antigens (Chanzu and Ondondo 2014; Arunachalam et al. 2020; Tay et al. 2020). Therefore, it is necessary to maintain a balance between immune cell numbers and cytokine levels to ensure healthy aging.

Edible insects are a sustainable bioactive peptide source due to their high protein content (Min et al. 2016; Ha et al. 2022). Among them, the darkling beetle (Tenebrio molitor), specifically its larvae, commonly known as mealworms, serves as a viable source of bioactive peptides and is widely consumed (Chung et al. 2013; Ha et al. 2022). After conducting various preliminary safety studies, *Tenebrio molitor* larvae (TML) was administered to participants at a dose of 3,000 mg/kg/day with observed non-toxicity, and TML was acknowledged as a new food ingredient by the Korean Food and Drug Administration in 2014 (Yoo et al. 2013; Min et al. 2016; Sim et al. 2020). TML is also recognized as a source of high-quality proteins and essential fatty acids in food (Kang et al. 2017; Ha et al. 2022). TML is known for its high essential amino acid content compared to other edible insects (Baek et al. 2017). During their larval stage, they contain abundant unsaturated fatty acids, including higher levels of oleic and linoleic acids than those found in beef (Baek et al. 2017). Unsaturated fatty acids lower low-density lipoprotein (LDL) cholesterol levels that are strongly linked to cardiovascular diseases, while increasing the production of high-density lipoprotein (HDL) cholesterol (Barter et al. 2007). Regarding the efficacy of HDL cholesterol in cardiovascular disease prevention, an increase of 1 mg HDL per deciliter (dL) can reduce the risk of coronary artery disease by 2–3% (Jeon et al. 2015).

Previous research has highlighted the pharmacological potential of TML, exhibiting their capabilities in activities such as countering Alzheimer’s disease, anti-cancer, anti-osteoporosis, and anti-inflammatory effects (Youn et al. 2014; Zielińska et al. 2017; Kim et al. 2019; Wu et al. 2020). Oil processing is advantageous for preserving the nutritional content of food and enriching it with healthful fats and vitamins compared to raw food (Errico et al. 2022). Similarly, fermentation processing enhances the nutritional content of food through microbial metabolites from secondary metabolism (Ha et al. 2022). Therefore, we conducted secondary processing to enhance the pharmacological effects of TML and mitigate any aversion associated with its insect.

Our research aimed to investigate processed TML's amino acid and fatty acid content to promote healthy aging. Additionally, we assessed changes in anti-obesity effectiveness and immune activity in an aging mouse model following the consumption of processed TML, confirming their potential as functional foods for anti-obesity or immune enhancement.

## Materials and methods

### Insects material

#### *Tenebrio molitor* larvae (TML) oil extraction

**Figure 1.**
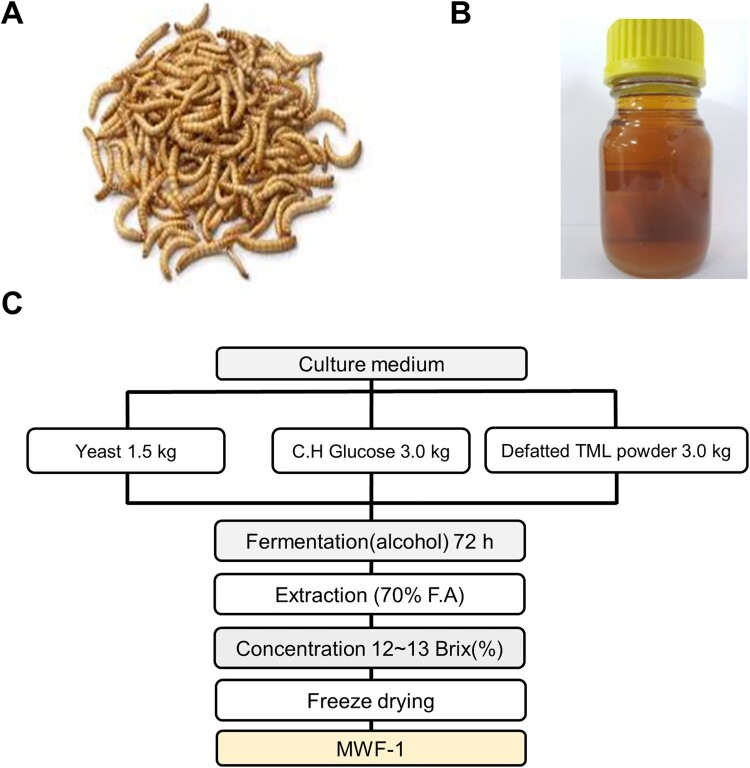
Processing details for producing oil and MWF-1 from *Tenebrio molitor* larvae (TML). Images of TML (A) and TML oil (B). C MWF-1 production process diagram.

An organic solvent (hexane) was used to extract TML oil by adding hexane to TML at a ratio of 10:1 (Figure 1(A)) and shaking for 24 h. The oil was concentrated using a rotary evaporator (Figure 1(B)).

#### Standardization of the manufacturing process of defatted mealworm fermented extract

KCTC 17299 was purchased from the Korean Cell Line Bank for the fermentation of *Tenebrio molitor* larvae (Figure 1(A)). Mealworm fermented extract (MWF-1) was prepared using defatted mealworm larval powder. The culture medium composition was yeast, defatted mealworm powder, and dextrose in a ratio of 1:2:2 (w/w) that was fermented with a *Saccharomyces cerevisiae* strain inoculum (1 × 10^6^ CFU/mL) at 30 ± 2°C for 72 h. The extracts were repeatedly extracted with 70% fermented alcohol by exhaustive maceration (3 × 7 L) at 85°C for 3 h using the reflux extraction method. The extracts were filtered through a Whatman No.2 filter paper. The filtered extracts were then concentrated using a rotary vacuum concentrator until they reached 12–13°Brix and were then freeze-dried. The freeze-drying samples were used for storage and experiments at −85°C after membrane filter sterilization (0.45 μm) (Figure 1(C)).

### Analysis of bound and free amino acids

To determine the amount of bound amino acids, 0.1 g of the sample was added to a tube, followed by 1 mL of 0.05% (v/v) 2-mercaptoethanol in 6 N HCl, and the contents were vortexed for 1 min. The tubes were filled with nitrogen gas to remove air, tightly capped, and placed in a dry oven at 105°C for 24 h to hydrolyze the samples. After hydrolysis, tubes were cooled in the dark for 30 min. The supernatant was transferred to a 50 mL volumetric flask, pure distilled water was added, and the mixture was filtered through a 0.2 µm syringe filter and transferred into a small glass vial for analysis.

To determine the amount of free amino acids, 1 g of the sample was transferred to a 50 mL volumetric flask, mixed with pure 0.02 N HCl, sonicated for 30 min, and centrifuged. After centrifugation, 10 mL of the supernatant was mixed with 25 mg of sulfosalicylic acid, incubated at 4°C for 4 h, filtered through a 0.2 µm syringe filter, and transferred into a glass vial for analysis.

High-performance liquid chromatography (HPLC; Agilent 1220, Agilent, Santa Clara, CA, USA) was performed by using a Proshell HPH C18 analytical column (150 mm × 4.6 mm, 4 µm; Agilent) and a variable wavelength detector, set to measure a wavelength of 338 nm. The column oven temperature was 40℃. Mobile phases A and B were 10 mM dibasic sodium phosphate,10 mM sodium tetraborate decahydrate = 1:1 (pH 8.2), and acetonitrile: methanol: water = 45:45:10 (v/v/v), respectively. Solvents A and B were run at a flow rate of 1.5 mL/min, using a gradient of 98% A (2% B) at 0 min, steady at 98% A for 1.9 min, decreased to 43% A for 16.2 min, decreased to 20% A for 0.5 min, steady at 20% A for 3.7 min, and then increased to 98% A for 0.9 min. The column was equilibrated with 98% A for 3.7 min before the next injection. O-phthalaldehyde and 3-mercaptopropionic acid in borate buffer (Agilent Technologies) were used for derivatization.

### Analysis of fatty acids

Sample (1 g) was mixed with 2 mL of 5% pyrogallol solution, 1 mL of internal standard solution (IS: C11:0 triundecanoin, 5 mg/mL in isooctane), 10 mL of 8.3 M HCl and heated using a shaking water bath (80°C, 75 min) for acid decomposition. The mixtures were cooled on ice, 15 mL of diethyl ether was added, and the mixture was centrifuged. The supernatant (ether layer) was collected and passed through an anhydrous sodium sulfate column to remove moisture and impurities. Then, 15 mL of petroleum ether was added, and the mixture was centrifuged. The supernatant (ether layer) was collected and passed through an anhydrous sodium sulfate column to remove moisture and impurities. The solvent was removed by using nitrogen gas to remove the ether layer and mixed with 0.5 N methanolic NaOH (2 mL). The mixture was heated using a water bath (85°C, 10 min) and cooled down to room temperature. BF3 methanol (2 mL) was added and heated using a water bath (85°C, 10 min). The mixture was cooled to room temperature, and isooctane (2 mL) and saturated NaCl (5 mL) were added. The mixture was then vortexed vigorously. The isooctane layer was transferred into a 2 mL vial. The aliquots (1 μL) of the extracts were analyzed by gas chromatography (Agilent Technologies 7890A, Santa Clara, CA, USA) by employing an autoinjector and a mass spectrometry (MS) detector using a fused-silica capillary column (Supelco SP-2560, 100 m × 0.25 mm i.d.; Supelco, Bellefonte, PA, USA). The injector and detector temperatures were 225°C, and 285°C, respectively. The oven was heated to 100°C and held for 4 min. Then, the oven temperature was increased to 240°C at the rate of 3°C /min, holding for 15 min at the final temperature. The carrier gas was helium, and the total gas flow rate at the inlet was 0.6 mL/min in the split mode (200:1).

### Experimental animals

C57BL/6J mice (7 and 28 weeks old, Male) were purchased from the Korean Basic Science Institute (KBSI). The animals were housed in a controlled environment (22 ± 2°C, 12 h light/12 h dark cycle, and humidity 50 ± 5%) in polycarbonate cages and fed a standard animal diet with water. The sample size for each experiment was determined based on previously published differences between control and old animals, ensuring sufficient statistical power while using the minimum necessary number of mice (Elabd et al. 2014). To investigate the effects of TML oil and MWF-1 on obesity, mice were divided into four groups: young group (*n* = 3, 8 weeks, distilled water 5 mL/kg), aged group (*n* = 3, 29 weeks, distilled water 5 mL/kg), TML oil group (*n* = 3, 29 weeks, TML oil 5 mL/kg), and MWF-1 group (*n* = 3, 29 weeks, MWF-1 500 mg/kg). TML oil and MWF-1 were orally administered three times per week for 6 weeks, and their body weights were recorded weekly. At the end of the diet intervention period, the mice were euthanized and blood and spleen samples were collected for subsequent biochemical and morphological analyses. The serum was collected and stored at −80°C until further use.

### Dual-energy X-ray absorptiometry (DEXA) analysis

The mean body fat density of the total body and abdominal cavity region (%) of each mouse was determined using dual-energy X-ray absorptiometry (DEXA; InAlyzer, EDIKORS, Korea) at the Korean Basic Science Institute (KBSI, Gwangju Center) on the final day of diet intervention period of the experiment.

### Biochemical analysis

Serum was obtained by centrifuging the blood at 3,000 rpm for 5 min. Serum triglyceride (TG) and total cholesterol (TCHO) levels were analyzed using an automated serum analyzer (FUJI DRI CHEM NX3500i; Fujifilm, Tokyo, Japan).

### Flow cytometry

Splenocytes were obtained from the spleens of mice in the four groups. The cells were then blocked with anti-mouse CD16/32 (Mouse BD Fc block; BD Biosciences, Franklin Lakes, NJ, USA) for 30 min on ice. After blocking, cells were immune-stained with anti-mouse CD4-FITC (BD Biosciences), CD8-PE (BD Biosciences), CD8-APC (BD Biosciences), NK1.1-FITC (BD Biosciences), CD19-FITC (BD Biosciences), IgM-Biotin (BD Biosciences), Streptavidin-PE-Cy5 (BD Biosciences), CD44-PE (BD Biosciences), Thy1.2-PE-Cy7 (BD Biosciences), and I-Ad-PE (BD Biosciences). The cell population was then gated using a FACS Canto II flow cytometer (BD Biosciences, San Diego, CA, USA). The data were analyzed using FlowJo software ver. 9.1 (Tree Star, Ashland, OR, USA).

### Enzyme-linked immunosorbent assay (ELISA)

Splenocytes of the young group, aged group, TML oil group, and MWF-1 groups were cultured in RPMI-1640 medium and stimulated with Concanavalin A (ConA, 1 µg/mL) or lipopolysaccharide (LPS, 1 µg/mL) for 24 h. Subsequently, the culture supernatant was assayed according to the manufacturer’s instructions (Lee et al. 2016).

### Proliferation assay

Splenocytes (C57BL/6 22weeks old, Male, 5 × 10^5^ cells/well) were seeded in 96-well plates, treated simultaneously with LPS (1 μg/mL) or Concanavalin A (1 μg/mL) and MWF-1 (100 μg/mL), and incubated (37°C, 5% CO_2_) for 24 h. CCK-8 reagents (Cell countingkit-8, Dojindo, Kumamoto, Japan) were used to assess cell proliferation. The optical density (O.D.) in 450 nm wavelength was evaluated by a Microplate reader (Versa max, Molecular Devices, CA, USA).

### Statistical analysis

The results are presented as means ± SEM (Standard Error Mean), and the statistical probability significance was established at the *P* < 0.05 level. Statistical differences between the groups were analyzed using SPSS ver. 27 software (SPSS, Chicago, IL, USA), using Tukey's test new multiple range post-hoc test.

## Results

### Bound and free amino acid contents

The bound amino acid contents during fermentation at 0 and 72 h (MWF-1) are shown in Table 1. A total of 15 amino acids were identified, including eight essential amino acids (Histidine, Threonine, Valine, Methionine, Phenylalanine, Isoleucine, Leucine, and Lysine) and seven non-essential amino acids (Aspartic acid, Glutamic acid, Serine, Glycine, Arginine, Alanine, and Tyrosine). Total bound amino acid content before fermentation (0 h value: 216.55 mg/g) was enhanced 2.1-fold after fermentation (72 h value: 448.59 mg/g; MWF-1) (Table 1). Individual amino acid-fold enhancement values were as follows: Valine (2.2-fold), Methionine (2.3-fold), Phenylalanine (2.0-fold), Isoleucine (2.0-fold), Leucine (2.1-fold), and Lysine (and 2.7-fold).

**Table 1.** Bound amino acid content of MWF-1 (72 h fermentation).

	Fermentation 0 h (mg/g)	MWF-1 (mg/g)
Aspartic Acid	23.34 ± 0.64	47.15 ± 2.03
Glutamic Acid	36.48 ± 0.47	87.72 ± 3.24
Serine	10.89 ± 0.05	22.35 ± 0.61
Histidine	9.16 ± 0.31	17.36 ± 1.50
Glycine	13.31 ± 0.15	28.58 ± 0.32
Threonine	9.78 ± 0.22	20.76 ± 0.94
Arginine	11.80 ± 0.44	11.92 ± 0.21
Alanine	18.67 ± 0.09	37.79 ± 1.16
Tyrosine	6.63 ± 0.44	4.32 ± 0.14
Valine	16.26 ± 0.13	35.53 ± 1.48
Methionine	1.31 ± 0.98	2.96 ± 0.16
Phenyalanine	9.97 ± 0.11	20.32 ± 0.62
Isoleucine	12.09 ± 0.12	23.80 ± 0.84
Leucine	18.09 ± 0.12	37.46 ± 0.77
Lysine	18.78 ± 0.20	50.59 ± 1.22
Total	216.55 ± 2.40	448.59 ± 14.82

The free amino acid contents during fermentation at 0 and 72 h (MWF-1) are shown in Table 2. The total free amino acid content before fermentation (0 h value: 6977.84 mg/100 g) was enhanced 2.7-fold after fermentation (72 h value:18542.56 mg/100 g; MWF-1) (Table 2). Among the amino acids whose levels were enhanced after fermentation, valine (3.3-fold), methionine (3.0-fold), phenylalanine (3.5-fold), isoleucine (2.5-fold), leucine (2.5-fold), and lysine (5.8-fold) are anti-obesity amino acids that prevent the conversion of carbohydrates into neutral fats and their accumulation in the body (Tables 1 and 2).

**Table 2.** Free amino acid content of MWF-1 (72 h fermentation).

	Fermentation 0 h (mg/100 g)	MWF-1 (mg/100 g)
Aspartic Acid	389.63 ± 10.67	768.62 ± 47.18
Glutamic Acid	658.32 ± 15.61	1946.78 ± 87.81
Serine	363.78 ± 8.57	915.69 ± 35.61
Histidine	203.53 ± 1.63	930.31 ± 48.56
Glycine	198.54 ± 4.01	944.36 ± 19.60
Threonine	272.48 ± 7.55	767.47 ± 40.09
Arginine	583.45 ± 8.76	85.18 ± 1.07
Alanine	931.65 ± 18.30	2347.21 ± 93.16
Tyrosine	381.47 ± 7.24	19.60 ± 1.08
Valine	609.87 ± 11.36	2028.69 ± 86.83
Methionine	106.72 ± 3.41	323.65 ± 11.19
Phenylalanine	395.57 ± 4.31	1403.98 ± 56.41
Isoleucine	565.83 ± 9.46	1433.97 ± 56.30
Leucine	917.90 ± 14.47	2322.06 ± 64.86
Lysine	399.09 ± 3.23	2304.98 ± 78.46
Total	6977.84 ± 119.10	18542.56 ± 725.61

Branched-chain amino acids (BCAA), including valine, isoleucine, and leucine, account for 35% of the essential amino acids in muscle proteins. These amino acids are oxidized in skeletal muscles and serve as a source of muscle energy (Wagenmakers et al. 1990). In addition, BCAA can prevent loss of muscle mass during body weight loss programs using diet or exercise and is also necessary for lymphocyte growth, proliferation, and activation of cytotoxic T lymphocytes (Churchward-Venne et al. 2013; Monirujjaman 2014).

### Fatty acid composition

Dietary fat is essential for improving imbalanced dietary habits in the elderly (Wahlqvist and Saviage 2000; Lands 2005). In our study, we assessed the types and quantities of fatty acids in TML oil and MWF-1 that are processed products of TML. TML oil contains nine types of saturated fatty acids (22.122 g/100 g) and five types of unsaturated fatty acids (73.978 g/100 g; Table 3). The MWF-1 fatty acid composition is expected to have an anti-obesity effect owing to its high octadecenoic acid content (48.008 g/100 g), a monounsaturated fatty acid known to reduce the risk of developing arteriosclerotic diseases, such as heart disease and cerebrovascular disease, by lowering cholesterol levels in the blood (Strøm et al. 2012). Conversely, MWF-1 contains a total of 21 types of fatty acids, including 15 saturated fatty acids (0.624 g/100 g) and six unsaturated fatty acids (0.818 g/100 g; Table 4). MWF-1 is expected to have an anti-obesity effect owing to its high octadecenoic acid content (0.405 g/100 g).

**Table 3.** Fatty acid content of TML oil.

Fatty acids	TML oil (g/100 g)
Capric acid (C10:0)	0.038
Lauric acid (C12:0)	0.292
Tridecanoic acid (C13:0)	0.045
Myristic acid (C14:0)	3.628
Pentadecanoic acid (C15:0)	0.089
Palmitic acid (C16:0)	15.735
Hexadecenoic acid (C16:1)	2.560
Margaric acid (C17:0)	0.134
Stearic acid (C18:0)	2.070
Octadecenoic acid (C18:1n9c)	48.008
Octadecadienoic acid (C18:2n6c)	23.270
Arachidic acid (C20:0)	0.092
Cetoleic acid (C20:1n9)	0.084
Eicosadienoic acid (C20:2)	0.056
∑SFA^a^	22.122
∑USFA^b^	73.978

^a^ Total saturated fatty acid = capric acid (C10:0) + lauric acid (C12:0) + tridecanoic acid (C13:0) + myristic acid (C14:0) + pentadecanoic acid (C15:0) + palmitic acid (C16:0) + margaric acid (C17:0) + stearic acid (C18:0) + arachidic acid (C20:0).

^b^ Total unsaturated fatty acid = hexadecenoic acid (C16:1) + octadecenoic acid (C18:1) + octadecadienoic acid (C18:2) + eicosadienoic acid (C20:2).

**Table 4.** Fatty acid content of MWF-1.

Fatty acids	MWF-1 (g/100 g)
Butyric acid (C4:0)	0.222
Caproic acid (C6:0)	0.002
Caprylic acid (C8:0)	0.001
Capric acid (C10:0)	0.002
Tridecanoic acid (C13:0)	0.001
Myristic acid (C14:0)	0.031
Pentadecanoic acid (C15:0)	0.001
Palmitic acid (C16:0)	0.230
Hexadecenoic acid (C16:1)	0.021
Margaric acid (C17:0)	0.003
Stearic acid (C18:0)	0.109
Octadecenoic acid (C18:1n9c)	0.405
Octadecadienoic acid (C18:2n6c)	0.379
Arachidic acid (C20:0)	0.013
Eicosenic acid (C20:1n9)	0.001
Linolenic acid (C18:3n3)	0.008
Heneicosanoic acid (C21:0)	0.001
Eicosadienoic acid (C20:2)	0.003
Behenic acid (C22:0)	0.006
Tricosanoic acid (C23:0)	0.001
Lignoceric acid (C24:0)	0.001
∑SFA^a^	0.624
∑USFA^b^	0.818

^a^ Total saturated fatty acid = butyric acid (C4:0) + caproic acid (C6:0) + caprylic acid (C8:0) + capric acid (C10:0) + tridecanoic acid (C13:0) + myristic acid (C14:0) + pentadecanoic acid (C15:0) + palmitic acid (C16:0) + margaric acid(C17:0) + stearic acid (C18:0)+ arachidic acid (C20:0) + heneicosanoic acid (C21:0) + behenic acid (C22:0) + tricosanoic acid (C23:0) + lignoceric acid(C24:0).

^b^ Total unsaturated fatty acid = hexadecenoic acid (C16:1) + octadecenoic acid(C18:1) + octadecadienoic acid (C18:2) + linolenic acid (C18:3) + eicosenic acid (C20:1) + eicosadienoic acid (C20:2).

### TML oil and MWF-1 regulate body fat in aging mice

**Figure 2.**
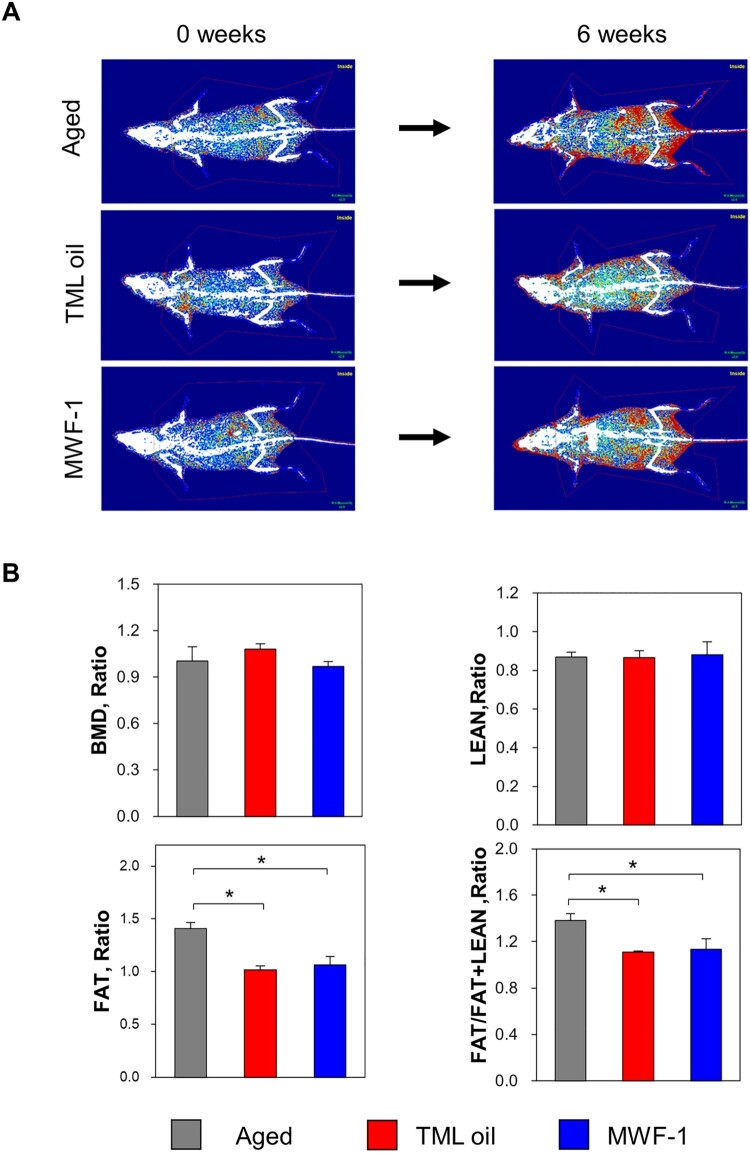
Effects of TML oil and MWF-1 on body weight and aging mouse fat accumulation. The bone density, fat mass (FAT), and muscle mass (LEAN) of aging mice were measured using DEXA (Dual-energy X-ray absorptiometry) after oral administration for 6 weeks with TML oil or MWF-1. Aged group: Oral administration of distilled water (5 mL/kg, *n* = 3). TML oil group: Oral administration of TML oil (5 mL/kg, *n* = 3). MWF-1 group: Oral administration of MWF-1 (500 mg/kg, *n* = 3). A DEXA image. Red, yellow, and blue indicate high-, intermediate-, and low-density fats, respectively. B Quantitative evaluation of bone mineral density (BMD), muscle mass (LEAN), and total body fat mass (FAT). The ratio was normalized to 0-day data. Data are presented as mean ± SD (*p*-values determined by one-way Tukey's test, *n* = 3, *p < 0.05 and **p < 0.01).

To confirm the effect of TML on body fat and muscle mass changes in aged mice, 29-week-old aged mice were administered TML oil or MWF-1 for 6 weeks, and their body fat distribution and muscle mass were evaluated using DEXA. As shown in Figure 2, DEXA's assessment of body fat and muscle mass revealed excessive fat accumulation in the abdominal and hip regions of the aged group (29-week-old) after 6 weeks of dietary intervention (Figure 2(A)). Quantitative analysis confirmed increased body fat mass (FAT) (Figure 2(B)). In contrast, oral administration of TML oil and MWF-1 suppressed the increase in body fat mass compared to the non-administered control group. Furthermore, quantitative analysis revealed no statistically significant changes in LEAN (muscle mass) (Figure 2(B)). However, body fat mass significantly decreased (*p *< 0.05) in the TML oil and MWF-1 groups compared to the aged group. In particular, the relative change in body fat mass compared to LEAN was lower in the TML oil and MWF-1 administration group than in the aged group.

#### TML oil reduces triglyceride levels in aged mice

**Figure 3.**
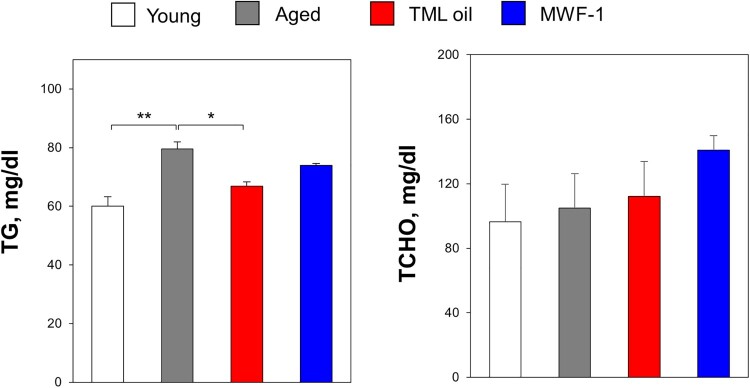
Effect of TML oil and MWF-1 on serum biochemical markers. The serum triglyceride (TG) and total cholesterol (TCHO) levels were determined in 29-week-old mice with or without the administration of TML oil (5 mL/kg) or MWF-1 (500 mg/kg) for 6 weeks. Quantification was done by using a diagnostic slide. Data are presented as mean ± SD (*p*-values determined by one-way Tukey's test, *n* = 3, *p < 0.05 and **p < 0.01).

After oral administration of TML oil and MWF-1, serum triglyceride and cholesterol levels were evaluated (Figure 3). In the case of young mice (8-week-old mice), the triglyceride levels (*p *< 0.05) were statistically significantly lower than those of aged mice (29-week-old mice). It was observed that triglyceride levels increased with aging; however, oral administration of TML oil significantly reduced triglyceride levels (*p *< 0.05), which decreased to the levels observed in young mice. The TML oil-derived reduction was statistically significant. No noticeable patterns of changes were observed in the 4 groups regarding TCHO.

### MWF-1 restores the reduced number of B cells in aged mice

**Figure 4.**
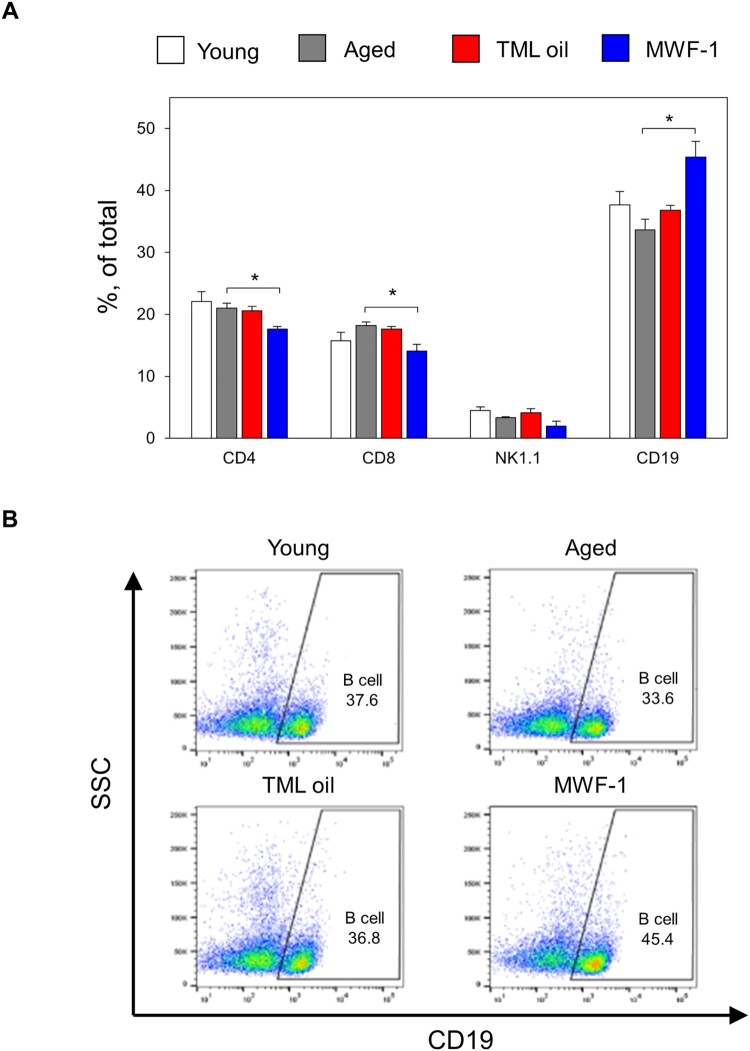
Effects of TML oil and MWF-1 on immune cell populations in aged mice. Splenocytes were analyzed by using flow cytometry. A Population of CD4+, CD8+, NK1.1+, and CD19+ cells in the spleen. B Proportion of B cells (CD19+) relative to total immune cells. Data are presented as mean ± SD (*p*-values determined by one-way Tukey's test, *n* = 3, *p < 0.05).

A decline in immunity related to aging leads to an increased susceptibility to infectious diseases, decreased vaccine effectiveness, and a higher risk of autoimmune diseases (Matsuzawa et al. 2011; Blanco et al. 2018). This is due to a decrease in the number and function of white blood cells (Rego et al. 1998). To explore the impact of TML and MWF-1 on immune function in aged mice, we collected splenocytes after the oral administration of TML oil and MWF-1 in such mice. We assessed the immune cell populations through flow cytometry analysis. We observed no significant difference in the percentage of helper T cells (CD4+), cytotoxic T cells (CD8+), natural killer cells (NK1.1+), and B cells (CD19+) in the TML oil-treated groups (Figure 4(A)). However, the number of B cells (CD19+) showed a significant increase (*p *< 0.05) in the MWF-1 group (45.4%) compared with the aged group (33.6%) (Figure 4(B)). The effects of MWF-1 were also consistent in *in vitro*. Proliferation assays with aged mouse splenocyte cells showed that while there was a 1.4-fold increase upon LPS stimulation, LPS + MWF-1 treatment led to a 1.9-fold increase (Figure S1). However, no significant differences were observed in ConA stimulation and ConA + MWF-1 treatment (Figure S1). Nonetheless, flow cytometry analysis revealed a 1.2-fold increase in the expression of CD44, a cell adhesion molecule expressed during the early activation of T cells, in CD4 T cells (Thy1.2+ CD4+) (Figure S2), indicating an improvement in T cell activation in aged mice. IgM expression in B cell activation is a marker for assessing the early stages of the humoral immune response and function in relation to the effectiveness of B cell activation against infections or diseases (Monzó et al. 2023). In flow cytometry analysis, MWF-1 enhanced IgM expression in aged B cells by 1.9-fold compared to IgM expression upon LPS stimulation alone (Figure S3). This enhancement signifies an improved differentiation into plasma cells, underscoring the immunomodulatory potential of MWF-1.

### TML oil and MWF-1 restore cytokine levels in immune cells in aged mice

**Figure 5.**
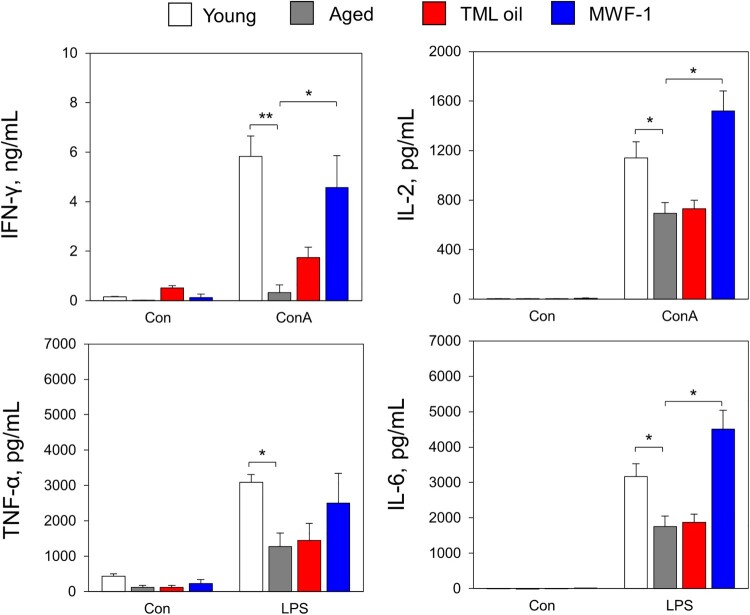
Effect of TML oil and MWF-1 on the production of cytokine in splenocytes. The splenocytes were treated with LPS (1 µg/mL) or ConA (1 µg/mL), and the cytokines released into the cell culture medium were measured using ELISA kits. IFN-γ secretion, IL-2 secretion, TNF-α secretion, and IL-6 secretion. Data are presented as mean ± SD (*p*-values determined by one-way Tukey's test, *n* = 3, *p < 0.05 and **p < 0.01).

To evaluate the production of cytokines by immune cells, we obtained splenocytes after the administration of TML oil and MWF-1 for 6 weeks, stimulated splenocytes with LPS and ConA, and measured the cytokine levels by ELISA. In the case of aged mice, a statistically significant decrease (*p *< 0.05) in cytokine (IL-6, TNF-α, IL-2, and IFN-γ) secretion was observed compared to young mice (Figure 5). In mice administered MWF-1 orally, there was an increase in the release of all cytokines, which were recovered to the levels of splenocytes cultured from young mice. Notably, the IFN-γ IL-2 and IL-6 levels were higher in the MWF-1 group cultured splenocytes than those cultured from young mice.

## Discussion

As the proportion of the elderly continues to grow, the proportion of individuals over 60 is projected to reach 21% by 2050 (Sarah 2014). Obesity is a significant health issue that affects the elderly population (Zoico et al. 2019; Kwon et al. 2023). The prevalence of obesity is reported to be approximately 11% and tends to increase with age (Marinos 2001; Gao et al. 2021; Ji et al. 2022). According to research findings, individuals between the ages of 60 and 75 years change in body composition, including an overall increase in body fat, a decrease in peripheral subcutaneous fat, and a predominant accumulation of visceral fat (Weiskopf et al. 2009; Batsis and Villareal 2018; Koliaki et al. 2019). These alterations in body composition are closely linked to age-related factors, such as insulin resistance, declining levels of thyroid hormones, and hormonal fluctuations, including sex hormones (Batsis and Villareal 2018). Consequently, they contribute to the expansion of adipose tissue and the infiltration of visceral fat (Batsis and Villareal 2018).

The second significant issue is immune aging which typically begins in healthy adults around the age of 50 (Weiskopf et al. 2009). Immune aging is characterized by a decline in the ability to respond to new pathogens because of reduced numbers of naïve B cells while memory B cells fill up the immune space (Weiskopf et al. 2009). Aging B cells also have a diminished ability to undergo somatic hypermutation, resulting in weaker antibody responses against infectious agents. Additionally, age-related changes in B cells are associated with decreased expression of CD23, CD21, and CD35 (Yeo et al. 2001; Ratliff et al. 2013). Aging B cells tend to accumulate in the bone marrow and inhibit B cell lymphopoiesis in aged mice (Allman and Miller 2005; Ratliff et al. 2013). T cells also experience a depletion of the pure T cell sub-population in individuals aged 60 and above (Ratliff et al. 2013; Salam et al. 2013). This depletion is accompanied by increased T cell immunoglobulin and immunoreceptor tyrosine-based inhibitory motif domain expression that contributes to T cell exhaustion (Salam et al. 2013). Therefore, dietary strategies should be implemented to prevent obesity and enhance the immune function in old age.

In the present study, we investigated the impact of processed TML oil and MWF-1 intake on obesity and immune responses in aging mice. We included both amino and fatty acids as part of our evaluation. First, our data revealed that processed MWF-1 is rich in amino acids such as valine, leucine, isoleucine, phenylalanine, methionine, and lysine, which contribute to body fat oxidation. Additionally, we investigated amino acids involved in lipase generation, such as arginine, threonine, alanine, and arginine, and found them abundant in MWF-1. These results suggest that TML oil and MWF-1 not only reduced age-related weight loss in aging mice but also effectively suppressed the levels of triglyceride, a marker of aged obesity, by treating with TML oil. Second, we also evaluated the fatty acid content of TML oil and MWF-1 to determine the nutritional composition of processed TML. Fats are essential nutrients in the human diet, and understanding the types and quantities of fatty acids in TML-based products can be particularly beneficial for improving the diet of elderly people with imbalanced dietary patterns. These TML-based products are expected to have an anti-obesity effect due to their high octadecenoic acid content (TML oil: 48.008 g/100 g, MWF-1: 0.405 g/100 g). Octadecenoic acid accounts for approximately 64.89% (TML oil) and 49.51% (MWF-1) of the unsaturated fatty acids. Third, we confirmed the effects of TML on obesity in aged mice. A remarkable discovery was the significant reduction in body fat mass but not in muscle mass. These findings suggest that TML-based products may contribute to healthy aging by controlling age-related changes in body composition.

Of particular interest among these results is the observation that TML oil and MWF-1 restored the immune responses in splenocytes derived from aging mice to levels comparable to those observed in splenocytes of young mice (8-week-old) following antigen stimulation. In particular, a significant increase in B cell numbers was observed with the consumption of MWF-1. Furthermore, the administration of TML oil and MWF-1 showed the restoration of cytokine levels, especially the induction of IFN-γ secretion in aging mice.

In summary, our study has confirmed the enhanced amino acid and free fatty acid content of TML-based products, such as TML oil and MWF-1, and has demonstrated their potential to be used as dietary supplements with positive implications for healthy aging by regulating immune enhancement and changes in body composition in aged mice. However, further experiments are necessary to elucidate the precise mechanisms induced by TML oil and MWF-1 to bring about immune enhancement during aging.

## Supplementary Material

Supplementary Material

## Data Availability

Data are contained within the article.

## Abbreviations

**Abbreviations:** BMD: Bone mineral density; ConA: Concanavalin A; DEXA: Dual Energy X-ray Absorptiometry; IL-1 β: Interleukin-1β; IL-2: Interleukin-2; IL-6: Interleukin-6; IFN-γ: Interferon-γ; LPS: Lipopolysaccharide; P.O.: Oral administration; ROI: Region of interest; TCHO: Total cholesterol; TG: Triglyceride; TNF-α: Tumor necrosis factor-α; MWF-1: Mealworms fermented extract-1
