# Supplementary material for: Anti-obesity and immunomodulatory effects of oil and fermented extract dried from Tenebrio molitor larvae on aged obese mice
Source: Anim Cells Syst (Seoul). 2024 Jul 13;28(1):340–52. doi: 10.1080/19768354.2024.2374547 (PMC11249154; doi:10.1080/19768354.2024.2374547)
Supplement: Supplementary Material [file TACS_A_2374547_SM0638.docx]

**Supplementary Information**

**Anti-obesity and immunomodulatory effects of oil and fermented extract dried from *Tenebrio molitor* larvae on aged obese mice**

Seul-Ki Mun^1,†^, Chang Joo Jang^1,†^, Semi Jo^1†^, Si-Hyoun Park^1†^, Hyun Bo Sim^1^, Sonny C. Ramos^1^, Hyeongyeong Kim^1^, Yu-Jeong Choi^1^, Dae-Han Park^1^, Kyung-Wuk Park^2^, Beom-Gyun Jeong^2^, Dae Heon Kim^1,3, *^, Kyung-Yun Kang^2,*^, Jong-Jin Kim^1,*^

^1^ Department of Biomedical Science, Sunchon National University, Suncheon 57922, Republic of Korea; motomoto1210@naver.com (S.-K.M); wqavcb@naver.com (J.J.C.); tpal9152@naver.com (S.J.); psh2992@naver.com (S.-H.P.); kokonun3@naver.com (H.B.S.); ynnosomarc@gmail.com (S.C.R); rlaguddud78@naver.com (H.K.); pskd357@naver.com (Y.-J.C.); carbdsinc@naver.com (D.-H.P.).

^2^ R&D team, Suncheon Research Center for Bio Health Care, Suncheon 57962, Republic of Korea; uk988446@sbrc.kr (K.-W.P.); fusionchef@sbrc.kr (B.-G.J).

^3^ CCRIPO Inc., Daejeon 34014, Republic of Korea

^†^ These authors contributed equally to this work.

^*^ Correspondence: dheonkim@sunchon.ac.kr (D.H.K.); nms-kang@sbrc.kr (K.-Y. K.); kimjj@scnu.ac.kr (J.-J.K.); Tel: +82-61-750-3752

**Supplementary Figures**


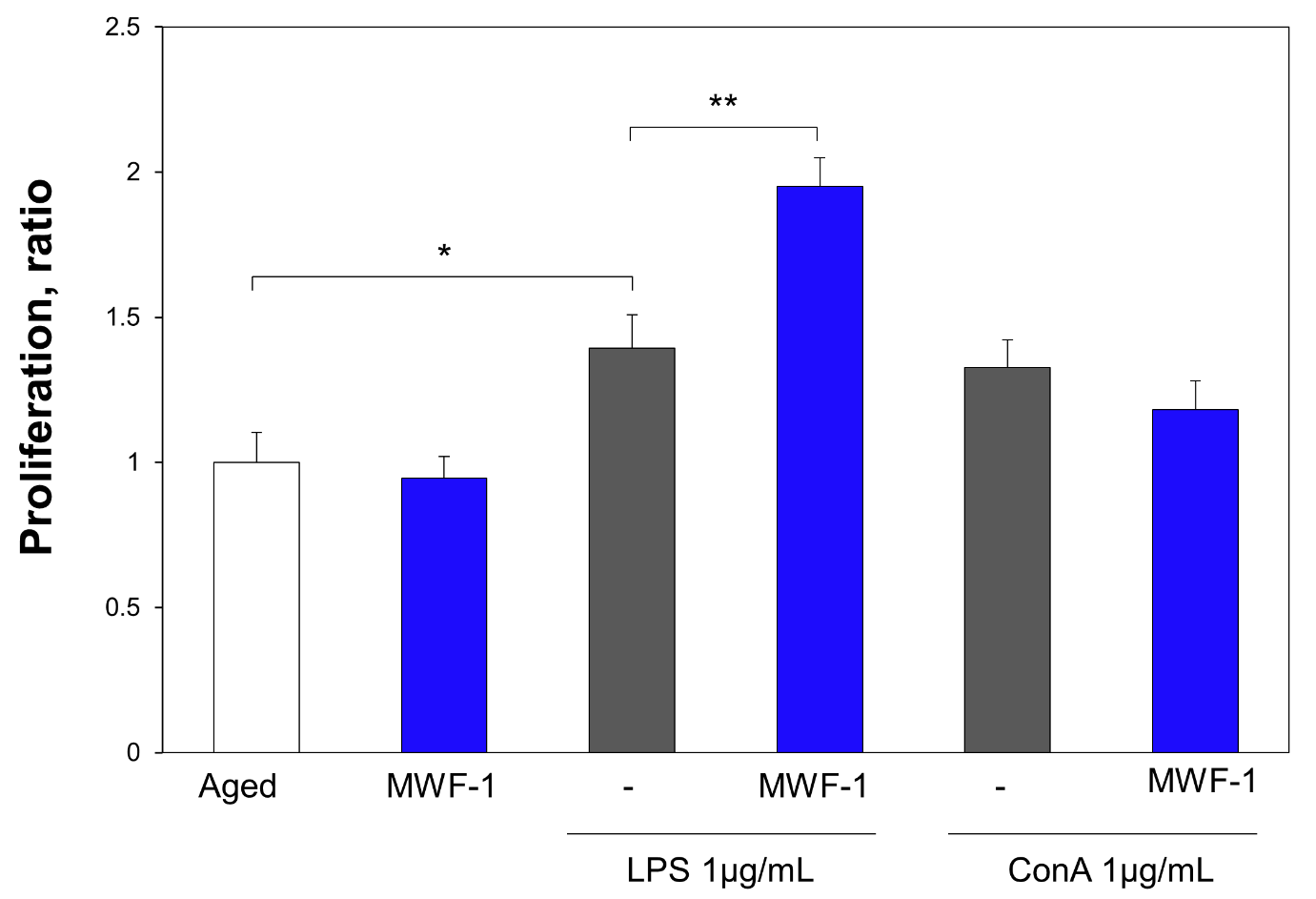


**Figures S1. The proliferative effect of MWF-1 in aged splenocytes.** The cells (C57BL/6, 5 × 10^5^ cells/well) were stimulated with LPS (1 μg/mL) or ConA (1 μg/mL) and simultaneously treated with MWF-1 (100 μg/mL) for 24 h. Proliferation was measured using the cell counting kit-8. The aged group (media) was used to normalize the data. Values represent mean ± S.D (n=3, **p*<0.05, ***p*<0.01).


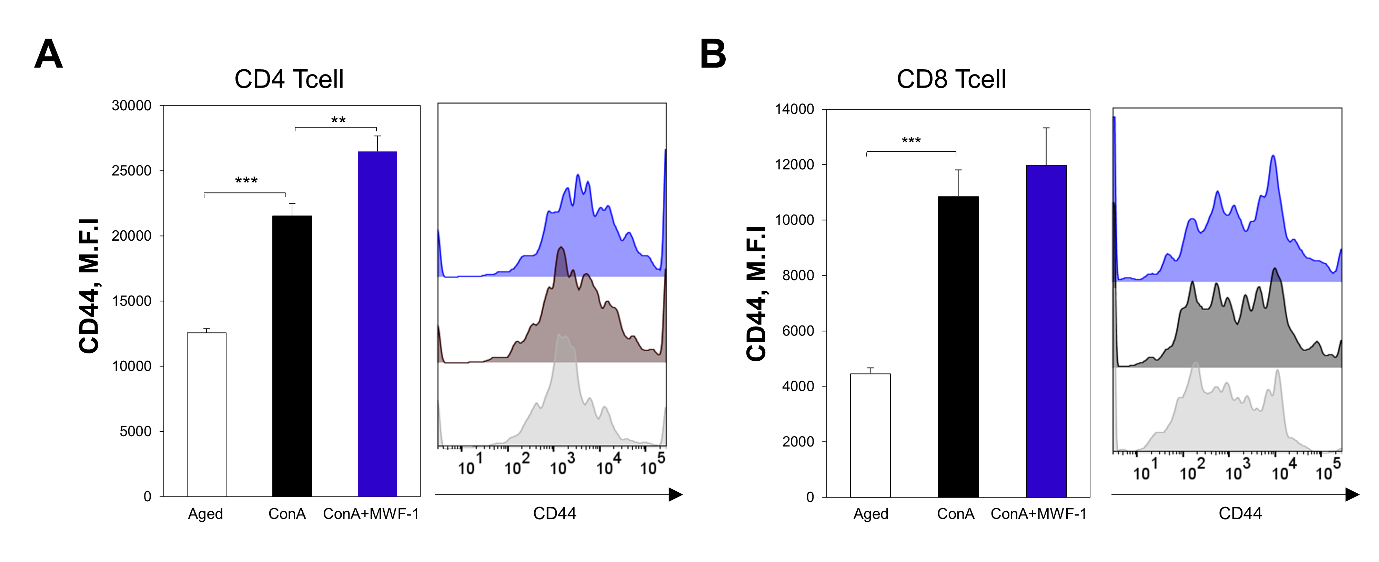


**Figures S2.** **Enhanced effect of MWF-1 on T Cell function.** C57BL/6 cells (5 × 10^5^ cells/well) were stimulated with ConA (1 μg/mL) and simultaneously treated with MWF-1 (100 μg/mL) for 24 hours. The cells were fluorescently stained with Thy1.2-PECy7, CD8-APC, and CD44-PE, and analyzed by flow cytometry using FlowJo software version 10.8.1. Data are presented as mean ± S.D. (***P*<0.01, ****P*<0.001).


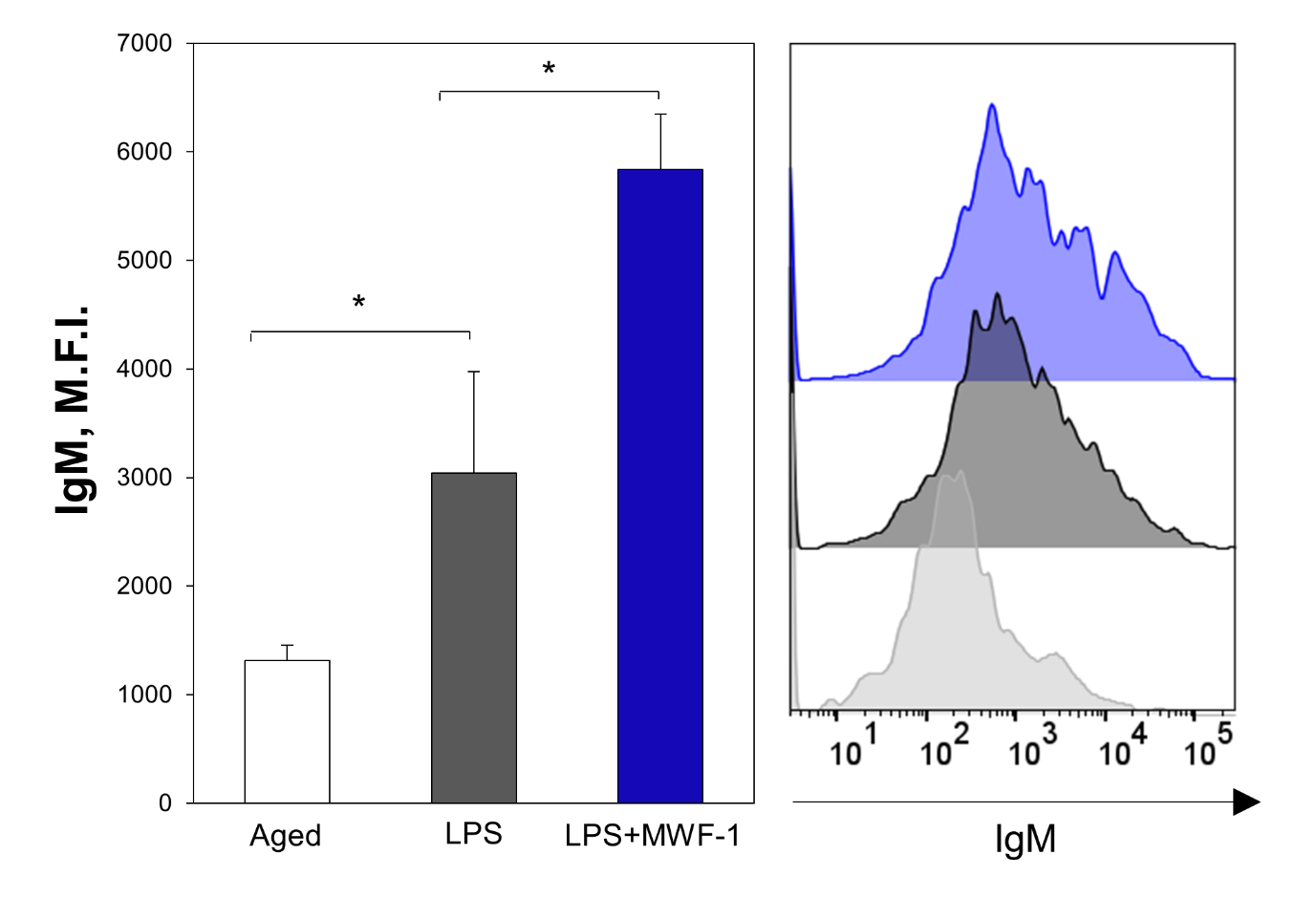


**Figures S3. Effect of MWF-1 on immunoglobulin expression of IgM on the CD19 - aged B cell.** C57BL/6 cells (5 × 10^5^ cells/well) were stimulated with LPS (1 μg/mL) and simultaneously treated with MWF-1 (100 μg/mL) for 24 hours. The cells were fluorescently stained with CD19-FITC, IgM-PE-Cy5, and analyzed by flow cytometry using FlowJo software version 10.8.1. Data are presented as mean ± S.D. (***P*<0.01, ****P*<0.001).
